# Current understanding of T cell immunity against SARS-CoV-2

**DOI:** 10.1186/s41232-022-00242-6

**Published:** 2022-11-29

**Authors:** Xiuyuan Lu, Sho Yamasaki

**Affiliations:** 1 Laboratory of Molecular Immunology, Immunology Frontier Research Center, Osaka University, Suita, 565-0871 Japan; 2 Epitope Analysis Team, Center for Advanced Modalities and DDS, Osaka University, Suita, 565-0871 Japan; 3 Department of Molecular Immunology, Research Institute for Microbial Diseases, Osaka University, Suita, 565-0871 Japan; 4 Center for Infectious Disease Education and Research (CiDER), Osaka University, Suita, 565-0871 Japan; 5 Division of Molecular Design, Research Center for Systems Immunology, Medical Institute of Bioregulation, Kyushu University, Fukuoka, 812-8582 Japan

**Keywords:** COVID-19, Infection, Vaccine, Adaptive immunity, Tfh, Variants, HLA, Cross-reactive T cell, HCoVs, Commensals

## Abstract

As an important part of adaptive immunity, T cells are indispensable in the defense against pathogens including viruses. SARS-CoV-2 is a new human coronavirus that occurred at the end of 2019 and has caused the COVID-19 pandemic. Nevertheless, most of the infected patients recovered without any antiviral therapies, suggesting an effective immunity developed in the bodies. T cell immunity responds upon SARS-CoV-2 infection or vaccination and plays crucial roles in eliminating the viruses and generating T cell memory. Specifically, a subpopulation of CD4^+^ T cells could support the production of anti-SARS-CoV-2 antibodies, and cytotoxic CD8^+^ T cells are also protective against the infection. SARS-CoV-2–recognizing T cells could be detected in SARS-CoV-2–unexposed donors, but the role of these cross-reactive T cells is still in debate. T cell responses could be diverse across individuals, mainly due to the polymorphism of HLAs. Thus, compared to antibodies, T cell responses are generally less affected by the mutations of SARS-CoV-2 variants. Up to now, a huge number of studies on SARS-CoV-2–responsive T cells have been published. In this review, we introduced some major findings addressing the questions in the main aspects about T cell responses elicited by SARS-CoV-2, to summarize the current understanding of COVID-19.

## Background

In the host defense against viral infection, adaptive immunity is the most effective component of the immune system. In adaptive immunity, neutralizing antibodies produced by humoral immunity target the free virus particles, preventing them from infecting other host cells. Meanwhile, cellular immunity managed by T cells has two major roles: cytotoxic T cells (mainly CD8^+^) attack the infected cells, stopping the ongoing viral proliferation; and helper T cells (CD4^+^) provide support to other parts of the immune responses.

Severe acute respiratory syndrome coronavirus 2 (SARS-CoV-2, also called 2019-nCoV previously) is the pathogen of human coronavirus disease 2019 (COVID-19). Since its emergence in 2019, SARS-CoV-2 has caused a worldwide pandemic that led to over 6 million deaths and almost 600 million infection cases within 3 years. Among all the other human coronaviruses (HCoVs), SARS-CoV-2 is genetically similar to severe acute respiratory syndrome coronavirus (SARS-CoV-1 or SARS-CoV) [1], which spread in 2003 and caused severe acute respiratory syndrome (SARS). Both SARS-CoV-1 and SARS-CoV-2 bind human angiotensin-converting enzyme 2 (ACE2) with their spike (S) proteins, using ACE2 as the receptor to enter human cells [2]. The understanding of immune responses against SARS-CoV-1 provided information for the prophylactic and therapeutic treatments against SARS-CoV-2 infection.

For the above reasons, anti-SARS-CoV-2 antibody responses were explored soon after the outbreak of COVID-19 and indeed detected in the sera of COVID-19 patients [3, 4]. Thus, the anti-SARS-CoV-2 antibody levels have been used as indicators of infection or efficacy of vaccination afterwards.

In contrast to the antibody response, the role of T cell response in host defense against SARS-CoV-2 has been less intensively investigated, probably due to the difficulties in sampling and measurement, as well as the complexities of T cell subpopulations and human leukocyte antigen (HLA)-restricted epitope-recognition mechanisms. Nevertheless, accumulating studies show that some T cells recognizing the SARS-CoV-2 antigens function in limiting and resolving the infection. This review aims to introduce the reports regarding the T cell response against SARS-CoV-2, for understanding the current situation and providing information for future studies of cellular immunity in COVID-19.

## Main text

### SARS-CoV-2 antigens elicit T cell responses

Given the nature of the SARS-CoV-2 pathogen, T cells were expected to respond to and eliminate the SARS-CoV-2 infection. As predicted, in most of the COVID-19 patients, SARS-CoV-2–activated CD4^+^ and CD8^+^ T cells could be detected [4, 5]. Various subpopulations of CD4^+^ T cells could be activated by SARS-CoV-2 antigens, including type 1 helper T cells (Th1), Th17, follicular helper T cells (Tfh), regulatory T cells (Treg), and CD4 cytotoxic T cells (CD4 CTL) [6]. T cells mainly recognize spike (S), membrane (M), nucleocapsid (N), non-structural protein 3 (nsp3, in ORF1ab), and ORF3a proteins in SARS-CoV-2 [5, 7]. Durability studies showed that SARS-CoV-2–specific memory T cells developed in some COVID-19 patients could last for at least 1 year [8, 9].

Similar to the convalescent patients who were naturally infected by the virus, donors who accepted SARS-CoV-2 vaccination also developed robust and durable T cell responses against the SARS-CoV-2 antigen [10–12]. Besides in the peripheral blood, antigen-specific T cells could also be detected in tissues like lymph nodes and mucosa after vaccination [13, 14]. T cell responses elicited by vaccination peaked at around 1 month after the 1st dose of vaccination and decreased with time after the peak, but reached a plateau at around 3–4 months [10, 14]. A 3rd booster dose of vaccine could significantly increase T cell responses, sometimes to a higher level than that developed after the 2nd dose [15, 16]. In convalescent patients, SARS-CoV-2 vaccination could induce T cell responses faster than in naïve individuals, but to a similar level [10]. SARS-CoV-2–specific T cells robustly express cytokines such as IFNγ, IL-2, TNF, and other cytokines and chemokines that are related to T cell functions [5, 6, 17–20]. Memory T cells induced by SARS-CoV-2 infection or vaccination could keep the potential of cytokine production for a long period [19, 20] and develop a stem cell memory T cell (T_SCM_) phenotype [8, 11, 21]. Updates about immunological memory against SARS-CoV-2 were reviewed recently [22].

T cell responses induced by SARS-CoV-2 vaccination were also investigated in the patients who received B cell depletion treatments. Although the development of T cells is considered to be independent of antibodies, several studies showed that patients who were treated with CD20 B cell-depleting therapy due to various diseases had more irresponsive cellular immunity to SARS-CoV-2 vaccination compared to healthy donors [23–25]. The mechanism of this phenomenon is still unclear, as some multiple sclerosis patients who accepted similar anti-CD20 therapy showed comparable or even augmented CD4^+^ and CD8^+^ T cell responses, besides compromised Tfh response [26]. Further studies are necessary to address this issue.

### T cell responses contribute to the host defense against SARS-CoV-2 infection

Along with the observations of T cell responses to SARS-CoV-2, growing evidence suggests that T cell responses are protective against SARS-CoV-2 infection. Tan et al. reported that COVID-19 patients with milder symptoms and earlier viral clearance tended to have more IFNγ-producing SARS-CoV-2–specific T cells that were induced at an early stage [19]. Patients with mild symptoms were more likely to have higher proportions of both CD4^+^ and CD8^+^ antigen-responsive T cells [17, 27]. Consistent with that, patients that were identified with diminished CD4^+^ T cells and CD8^+^ T cells in high-dimensional analyses tended to have higher mortality, regardless of the numbers of B cells [28]. COVID-19 patients with agammaglobulinemia (lacking B cells and antibody immunity) tended to have prolonged infection but milder symptoms [29–31].

Among all subsets of CD4^+^ T cells, Tfh cells are known to support antibody production that is pivotal in anti-viral immunity, and thus, their functions against SARS-CoV-2 were intensively investigated. Kaneko et al. showed that Tfh function and germinal center development were impaired in deceased COVID-19 patients [32], implying an important role of Tfh cells in the recovery from COVID-19. Consistent with that, the proportion of SARS-CoV-2–specific Tfh1 cells, a subpopulation of Tfh cells characterized with CXCR3^+^, correlated with the titers of anti-SARS-CoV-2 antibodies [33–35], suggesting that Tfh1 cells may positively contribute to the immune response against COVID-19.

CD8^+^ T cells are known for their cytotoxicity, which could kill the host cells when their TCRs recognize the antigens presented on the host cells. Instead of preventing infection from happening, cytotoxic T cells target the infected cells and restrain and eliminate an occurring infection. Besides the observations from COVID-19 patients mentioned above, data from non-human primates gave more evidence of the role of CD8^+^ T cells. Depletion of CD8^+^ T cells in macaques pre-immunized with a SARS-CoV-2 vaccine or living virus infection delayed the viral clearance in a later infection challenge [36, 37]. Another study in macaques immunized with a nasal vaccine developed using N, M, and envelope (E) proteins (that could minimize the influence of neutralizing antibodies) suggested that numbers of antigen-specific CD8^+^ T cells negatively correlated with the viral burden during infection challenge [38]. The above studies suggest profound functions of both CD4^+^ and CD8^+^ T cells in anti-SARS-CoV-2 immunity.

### HLA types and T cell responses against SARS-CoV-2

Unlike B cell receptors that can recognize their antigens directly, T cell receptors recognize epitopes that are processed and presented on major histocompatibility complex (MHC) molecules. MHC molecules in humans, the HLAs, are extremely polymorphic, which gives rise to the diverse T cell responses in different individuals.

However, some T cell epitopes restricted by prevalent HLAs could be recognized by T cells across different individuals [14, 39–42]. These restricting HLAs, including A*02, B*07:02, DPB1*04, and DRB1*15, could cover a large population worldwide [43]. Based on these observations, it is easy to hypothesize that possessing or lacking a certain HLA allele might influence the effectiveness of anti-SARS-CoV-2 immune responses. Indeed, there are many reports about the resistance or susceptibility to SARS-CoV-2 of donors who possess or lack certain HLAs [40, 44–48]. However, it is non-negligible that all these reports sampled less than 200 donors with similar religions or within a local region. In contrast, a study that analyzed over 3800 donors showed that there was no certain HLA bias for the susceptibility and severity to COVID-19 [49]. Possible reasons could be that some T cell epitopes can be presented on multiple HLAs [42], or the large variety of T cell epitopes reduced the difference between individuals with different HLAs. Further studies including larger cohorts are necessary to reach a conclusion on this issue.

### T cell responses against different SARS-CoV-2 variants

During the pandemic, SARS-CoV-2 keeps evolving. Up to now, there are 5 variants of concern (VOCs) of SARS-CoV-2 that have been or are circulating: Alpha (B.1.1.7), Beta (B.1.351), Gamma (P.1), Delta (B.1.617.2), and Omicron (B.1.1.529, including BA.1, BA.2, BA.3, BA.4, BA.5, and descendent lineages) [50]. These VOCs are either more infectious or could induce more severe symptoms than the ancestral strain. Among them, the Omicron variant has the highest infectivity, the largest number of mutations, and thus the highest possibility to escape from the established protectivity based on previous viral strains [51–53]. The Omicron variant started its spreading at the end of 2021 and replaced the former dominant strain, the Delta variant, with a very fast speed. In contrast to the previous VOCs that have less than 10 mutations in the S protein, the Omicron variant has more than 30 mutations, and almost half of them are located in the receptor binding domain (RBD). As predicted, these mutations in the S protein largely attenuated the protectivity of humoral immunity developed against previous strains [54, 55]. Nevertheless, several studies suggested that in donors who were vaccinated with ancestral S protein or infected by previous strains, the T cell responses against the Omicron variant were largely maintained compared to those against other variants [54–56]. Different types of exposure might show some differences in inducing the Omicron-resistant T cell responses, but the vaccine-induced S-recognizing T cells were generally maintained until 6 months after vaccinations [55]. On an epitope level, previously identified T cell epitopes in S and non-S proteins of SARS-CoV-2 are substantially conserved in VOCs including the Omicron variant [42, 54, 57]. In contrast to the S protein, the non-S proteins of SARS-CoV-2 have lower selection pressure from the humoral immunity of the host. Thus, the T cell epitopes located in non-S proteins are more likely to maintain antigenicity. Considering T cell epitopes vary between individuals because of diverse HLAs and TCR repertoire, it is possible that T cell immunity is more robust against SARS-CoV-2, including future variants. However, no definite prediction could be made about this issue.

### Cross-reactive T cells recognize SARS-CoV-2 and other antigens

SARS-CoV-2–reactive T cells exist in the peripheral blood and tonsil from donors unexposed to SARS-CoV-2 [5, 6, 18, 58–63]. Some of these studies analyzed samples collected before the COVID-19 pandemic [5, 6, 58, 59, 62, 63], excluding the possibility that the donors were exposed to SARS-CoV-2. One hypothesis of this phenomenon is that these SARS-CoV-2–cross-reactive memory T cells have been generated upon similar antigens derived from other coronaviruses. Previous to SARS-CoV-2, 6 HCoVs were identified. Among them, SARS-CoV-1 and the Middle East respiratory syndrome coronavirus (MERS-CoV) could cause respiratory syndromes and are more homologous to SARS-CoV-2 [1]. Le Bert et al. reported that SARS-CoV-2–recognizing T cells could be detected in convalescent SARS patients after 17 years [62]. However, SARS-CoV-1 and MERS-CoV were much less prevalent than SARS-CoV-2, suggesting that the cross-reactivities are unlikely to cover a wide range of the population. The other 4 human coronaviruses are HCoV-OC43, HCoV-HKU1, HCoV-NL63, and HCoV-229E, which are known to cause the common cold. The four common cold HCoVs are less homologous to SARS-CoV-2, but more widely circulating. Indeed, cross-reactive epitopes that could activate SARS-CoV-2–recognizing T cells were determined from common cold HCoVs [58, 64–66].

However, it is still undetermined whether common cold HCoVs-induced SARS-CoV-2–reactive T cells are beneficial in the host defense against COVID-19. It is reported that a SARS-CoV-2 peptide S816-830, which is relatively conserved in common cold HCoVs, could activate CD4^+^ T cells in 20% of SARS-CoV-2–unexposed donors and more infected or vaccinated donors, and thus, the authors concluded that the cross-reactive CD4^+^ T cells could be protective in COVID-19 [64]. In spite of that, another group showed that the same epitope activated CD4^+^ T cells in more donors who got breakthrough infections after vaccination than in vaccinated donors who had no breakthrough infection [66]. Furthermore, Bacher et al. reported that SARS-CoV-2–cross-reactive CD4^+^ T cells in unexposed individuals had low avidity, which is one of the characteristics of SARS-CoV-2–responsive CD4^+^ T cells in infected individuals with severe symptoms [61]. Although these cross-reactive CD4^+^ T cells could be activated by SARS-CoV-2 antigens and were expanded upon exposure to SARS-CoV-2, their role is still to be determined.

Besides the HCoVs, other commensals could also induce SARS-CoV-2–cross-reactive T cells [42, 67]. A public Tfh clonotype that could recognize SARS-CoV-2 S870-878 and a symbiotic bacterial antigen was detected in both healthy donors and COVID-19 patients, and had higher frequencies in patients with milder symptoms than in patients with severe symptoms, suggesting that this T cell clonotype was protective in COVID-19 [42]. Further studies are necessary to explore the characteristics of cross-reactive T cells and their roles against SARS-CoV-2.

### Ambiguous functions of several T cell subsets in COVID-19

As parts of the complex immune system, some T cell subsets seemed not to associate with protective immune responses or good outcomes in COVID-19 patients. It was reported that the proportion of CXCR3^–^ Tfh cells and a cytotoxic Tfh subpopulation negatively correlated with the anti-SARS-CoV-2 antibodies in COVID-19 patients [6, 33, 35], possibly because the Tfh response skewed to CXCR3^+^ subset, which contributes to anti-SARS-CoV-2 antibody production. SARS-CoV-2–recognizing CD4 CTL was likely to be another subset that implied adverse outcomes, as it showed increased proportion in the peripheral blood, draining lymph nodes and lungs from more severe patients [6, 68]. However, it is still unclear whether the responses of these T cell subsets are reasons or results of poor immune defense against SARS-CoV-2. More investigations are needed to elucidate these questions.

## Conclusion

During the more than 2 years of the COVID-19 pandemic, plenty of research was conducted, addressing almost every aspect of T cell response against SARS-CoV-2 infection. Consensus has been reached on some issues, such as T cell response could be protective against SARS-CoV-2 infection for a long duration, T cell epitopes in SARS-CoV-2 are relatively conserved, and cross-reactive T cells have existed before an individual gets exposed to SARS-CoV-2, as discussed in this review. However, answers to more questions are still obscure, such as the function of some minor T cell subsets including unconventional T cells [69], the role of cross-reactive T cells, and whether T cell response would be protective enough against future variants. To cease the COVID-19 pandemic, research on T cells and related immune responses will be continued. It has never been more important for immunologists in the COVID-19 era to conduct their studies cautiously and explain the results accurately without any misleading or exaggeration.

## Data Availability

Not applicable.

## Abbreviations

COVID-19: Coronavirus disease 2019
SARS-CoV-2: Severe acute respiratory syndrome coronavirus 2
SARS-CoV-1: Severe acute respiratory syndrome coronavirus
HCoV: Human coronavirus
Tfh: Follicular helper T
CTL: Cytotoxic T cell
HLA: Human leukocyte antigen
VOC: Variant of concern
